# 28. BEYOND VOICES: MULTISENSORY BODILY SELF DISTURBANCES ACROSS THE SCHIZOPHRENIA SPECTRUM

**DOI:** 10.1093/schbul/sby014.115

**Published:** 2018-04-01

**Authors:** Sohee Park

**Affiliations:** Vanderbilt University

## Abstract

**Overall Abstract:** Aberrant bodily self experiences are highly salient and disruptive to individuals with schizophrenia. Given that these self disturbances are already present at prodromal stage, persist throughout the course of illness, and impact functional outcome, they should be precisely targeted for intervention. However, in contrast to the prominence of auditory hallucination in schizophrenia research, bodily self disturbances have been largely neglected. This symposium aims to bridge this gap with new theoretical and experimental advances that allow us to capture the full extent of the phenomenology, and at the same time, mechanistically specify the etiology and nature of self disorders with an eye toward implementing new treatments.

Nelson & Sass will present their revised theory of self disturbances in schizophrenia that reconciles phenomenology with neuropsychological and empirical evidence. Raballo and Poletti will present a comprehensive analysis of recent developmental psychopathological studies of self disorders in the schizophrenia spectrum. Giersch and colleagues provide the crucial experimental evidence for the close link between weakened temporal expectancy and the disruption of continuous and unitary self in schizophrenia. Temporal expectancy helps to link and transform a chain of discrete events into our perceptual experience of continuous flow of time, but it is disrupted in schizophrenia. These results point toward potential intervention strategies. Park and colleagues present exteroceptive, proprioceptive and interoceptive contributions to abnormal mapping of bodily sensation and peripersonal space in the schizophrenia-spectrum and argue that the core problems may lie in prediction coding errors across multisensory systems. Potential intervention may lie in studying trained “sensorimotor experts” (athletes) who show precise and sharpened awareness of embodied sensations. The discussant (Ferri) will integrate these findings to highlight a new framework and multi-level approaches for understanding the etiologies of self disturbances in the schizophrenia-spectrum.

